# Association of *DNAH11* gene polymorphisms with asthenozoospermia in Northeast Chinese patients

**DOI:** 10.1042/BSR20181450

**Published:** 2019-06-20

**Authors:** Dongliang Zhu, Hongguo Zhang, Ruixue Wang, Xiaojun Liu, Yuting Jiang, Tao Feng, Ruizhi Liu, Guirong Zhang

**Affiliations:** 1 Center for Reproductive Medicine and Center for Prenatal Diagnosis, First Hospital of Jilin University, Changchun 130021, China; 2 Peking Medriv Academy of Genetics and Reproduction, Peking, China

**Keywords:** asthenozoospermia, DNAH11 gene, Male factor infertility, polymorphism

## Abstract

**Summary:** Reduced or no progressive sperm motility in the fresh ejaculate defines asthenozoospermia as one of the major causes of male infertility. The axonemal heavy chain dynein type 11 (*DNAH11*) gene encodes for one of the axonemal dynein heavy chain (DHC) family members and participates in assembling respiratory cilia and sperm flagella. Given the high degree of conservation of *DNAH11*, mutations could give rise to primary ciliary dyskinesia (PCD) and asthenozoospermia. To date, few studies have reported on the association between variants in *DNAH11* and asthenozoospermia. In the present study, 87 patients with idiopathic asthenozoospermia for variants in *DNAH11* were screened by using high-throughput targeted gene sequencing technology. Bioinformatics analysis was further assessed. We found compound heterozygous variants (c.9484-1 G>T, c.12428 T>C) of *DNAH11* detected in 1 of 87 patients. The variant c.9484-1 G>T was confirmed as a novel virulence variant which was predicted to affect splicing by Human Splicing Finder 3.1. And c.12428 T>C was predicted to be mildly pathogenic *in silico* analysis. We found that *DNAH11* polymorphisms display strong associations with asthenozoospermia, and may contribute to an increased risk of male infertility in Chinese patients.

## Introduction

Male factor infertility is a serious worldwide problem and accounts for 50% of cases of infertility [1]. The most common feature is abnormal semen quality. Four major semen anomalies, including azoospermia, oligozoospermia, asthenospermia and teratospermia, are present in almost 90% of infertile males [2]. In particular, asthenozoospermia is the second major cause of male infertility just inferior to oligozoospermia, and is characterized by reduced motility or the lack of progressive sperm motility in fresh ejaculates [3]. Many factors are related to asthenospermia, in which genetic factors are getting more and more attention. Previous studies have shown that the exonal single nucleotide polymorphism (SNP) rs1893316 in cation channel of sperm associated 1 *(CATSPER1), tektin-t* (R207H) gene polymorphism, prostate and testis 1 *(PATE1)* variant (A1423G), endothelial nitric oxide synthase *(eNOS)* (Glu298Asp) gene polymorphism may be associated with asthenozoospermia risk [4–7].

The *DNAH11* (OMIM: 603339) encodes for axonemal heavy chain dynein type 11, which is 353 kb in size, and it contains 82 exons yielding a 14 kb mRNA [8]. It has a high degree of conservation among humans, mice and even the protista such as *Chlamydomonas* [9,10]. In humans, the protein participates in assembling the outer dynein arms of the sperm axoneme, and is expressed in the trachea, lung and testis [11]; mutations in *DNAH11* can give rise to primary ciliary dyskinesia (PCD) in patients with normal axonemal ultrastructure [8,10–14]. Approximately 90% of men with PCD are diagnosed with asthenozoospermia and two reports have involved a link between *DNAH11* mutations and male infertility [12,15] (Table 1).

**Table 1:** Related reports on *DNAH11* mutations of humans in previous studies

Author	*DNAH11* mutations	Zygosity	Have a correlation with PCD?	Have a correlation with AZS?
Bartoloni et al. [10]	c.8554C>T	Homozygous	Yes	NM
Schwabe et al. [12]	c.12384C>G and c.13552_13608del	Compound heterozygous	Yes	Unclear
Zuccarello et al. [15]	c.9118A>G	Heterozygous	No	Yes
Pifferi et al. [13]	c.883-1G>A and c.4145G>A	Compound heterozygous	Yes	NM
	c.8135A>G and c.10284G>A	Compound heterozygous		
Knowles et al. [24]	c.4428C>T, etc.	Homozygous	Yes	NM
	c.12697C>T and c.12980T>C, etc.	Heterozygous		
Nakhleh et al. [28]	c.4520A>C and 9397G>A, 9203A>G	Homozygous	Yes	NM
Lucas et al. [25]	c.8719C>T and c.7793C>T	Compound heterozygous	Yes	NM
	c.6527C>A	Homozygous		
Raidt et al. [29]	Mutations	Compound heterozygous	Yes	NM
Boon et al. [26]	Mutations	Heterozygous/homozygous	Yes	NM
Dougherty et al. [14]	c.13183C>T	Homozygous	Yes	NM
	c.2753G>T and c.12796_12801delinsATA	Compound heterozygous		
	c.9304G>A c.4922C>G	Compound heterozygous		
Boaretto et al. [27]	c.11739+1 G>A, etc.	Homozygous	Yes	NM
	c.4775G>T and c.8589C>G, etc.	Compound heterozygous		
Shoemark et al. [30]	Mutations	Compound heterozygous	Yes	NM
Total	/	/	11	1

Abbreviations: PCD, primary ciliary dyskinesia; AZS, asthenozoospermia. NM, not mentioned.

Here, we screened 87 patients with asthenozoospermia for variants in *DNAH11* and discovered compound heterozygous *DNAH11* variants in one patient with asthenozoospermia using high-throughput targeted gene sequencing technology.

## Materials and methods

### Study population

Eighty-seven patients with asthenozoospermia [World Health Organization semen motility grades of progressive motility (PR) + nonprogressive motility (NP) <40%; PR <32% in fresh ejaculates] [16] including several patients with combined oligo and teratozoospermia were enrolled from 2011 May to 2016 April. Exclusion criteria included patients taking medications, leucocytospermia and presence of Mycoplasma and Chlamydia infection in semen, varicocele or systemic diseases, any history of cryptorchidism or orchitis, or an abnormal karyotype. Moreover, in all subjects, the presence of antisperm autoantibodies was excluded using human antisperm antibody from AsAb ELISA Kit (Beijing Beier Bioengineering Co., Ltd., Beijing, China). Serum reproductive hormones [follicle stimulating hormone (FSH), follicle stimulating hormone (LH) and testosterone (T)] were analyzed by automated electrochemiluminescence immunoassay analyzer (Cobas 6000-e601) (Roche Penzberg, Germany). Informed consent was provided by every patient before diagnosis. The present study was approved by the Ethics Committee of the First Hospital of Jilin University.

### Sample collection for genotyping

Aliquots of 5–10 ml of blood were collected from the patients into EDTA-coated anticoagulant tubes, and BloodGen Midi kits (Kangwei Century Biological Technology Co., Ltd., Beijing, P.R. China) were used for genomic DNA extraction.

### Targeted gene sequencing

Sequencing was carried out using the Illumina MiSeq platform (Illumina, San Diego, CA, U.S.A.) and an in-house targeted gene panel (Beijing Medriv Academy of Genetics and Reproduction, Beijing, P.R. China), which included the *DNAH11* gene. According to previous references and the OMIM database (http://www.omim.org), capture probes were established on the basis of asthenozoospermia-associated genes. Fragments with 3′/5′ linkers and tiny fragments of low quality were ruled out using Cutadapt (https://pypi.python.org/pypi/cutadapt) and FastQC (https://www.bioinformaticsbabraham.ac.uk/projects/fastqc/). The preprocessed clean reads were compared with the hg19 human reference sequence using BWA software (http://bio-bwa.sourceforge.net). Duplicated reads from the library and polymerase chain reaction preparations were removed with Picard tools. For single nucleotide variant (SNV) and Indel variants in the pre-processed sequence, the Genome Analysis Tool Kit (https://www.broad institute.org/gatk) was further employed in the analysis.

The calling quality was assessed with detection indexes as follows: 100% align rate >95%; 100% duplication rate <20%; rate of coverage ≥20× reading depth 92–99.99%; mean coverage of the target region >80×. Variants with population frequencies greater than 1% were filtered using 1000 Genomes (http://www.1000genomes.org/data), Exome variant Server (http://evs.gs.washington.edu/EVS/), Exome Aggregation Consortium (http://exac.broad institute.org/) and the dbSNP database (http://www.ncbi.nlm.nih.gov/snp). Except for synonymous variants, both rare and novel variants were reviewed for further investigation. For analysis of SNV, SIFT (http://sift-dna.org/) and PolyPhen-2 (http://genetics.bwh.harvard.edu/pph2/) algorithms were used to predict variants to damage protein function, and splicing harmfulness of any mutation close to splicing sites was also predicted with Human Splicing Finder 3.1 (http://www.umd.be/HSF3/).

## Results

*DNAH11* contains 82 exons and encodes a dynein protein comprising 4516 residues. The reference sequences for the present study are listed as NM_001277115.1(NCBI) and ENST00000409508(Ensembl)/Q96DT5 (UniProt). By sequencing, compound heterozygous *DNAH11* variants c.9484-1 G>T and c.12428 T>C were associated with one of the 87 patients with asthenozoospermia. The patient was 44 years old, had a 12-year history of infertility and no children. And he was diagnosed as asthenozoospermia (PR, 5.26%; total motility, 21.05%) with combined oligo-teratozoospermia (Table 2).

**Table 2:** General information of the patient with *DNAH11* gene variants

P1	Value	Normal reference value
Age (years)	44	
Duration of infertility	12	
Testicular size (left, ml)	15	
Testicular size (right, ml)	15	
Progressive motility (PR, %)	5.26	32
Total motility (PR + NP, %)	21.5	40
Sperm concentration (10^6^ per ml)	1.28	15
Normal spermatozoa (%)	NA	4
FSH (U/l)	14.1	1.5–12.4
LH (U/l)	5.6	1.7–8.6
T (nmol/l)	14.6	9.9–27.8
Clinical diagnosis	Oligoasthenoteratozoospermia	

Abbreviation: NA, not available.

**Figure 1:**
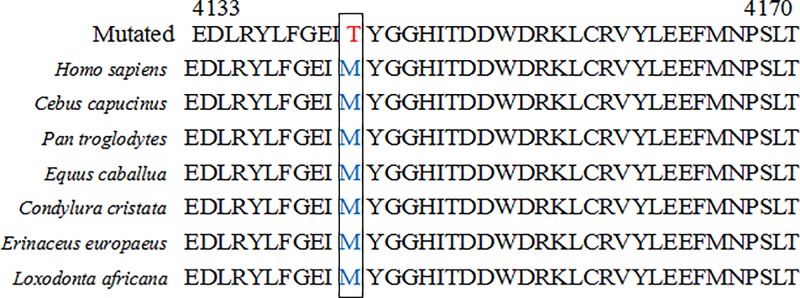
The sequence-alignment analysis of the *DNAH11* with the codon 4143M among seven different species

**Figure 2:**
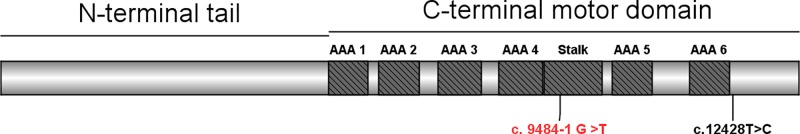
Location of c.9484-1 G >T and c.12428T>C in *DNAH11* domain structure The variant c.9484-1 G >T (in red) is absent from the 1000 Genomes and ExAC database.

The novel variant c.9484-1 G>T, located in a splice site of intron 57 of *DNAH11*, was predicted to affect splicing by Human Splicing Finder 3.1 (http://www.umd.be/HSF3/). The additional variant c.12428 T>C is located in exon 76 and results in a missense mutation (p.M4143T) in the C-terminal region of the protein. The amino acid 4143M is positioned in a highly conserved domain among seven species (Figure 1), and the variant c.12428 T>C was extremely rare (0.0000747). According to the Ensembl GRCh37 database (http://grch37.ensembl.org/index.html), the isolated variant in *DNAH11* (c.12428 T>C, rs751994566) could affect the protein function with a SIFT score of 0.01, but was forecast to be benign with PolyPhen (probabilistic score of 0.119). However, we further analyzed the missense variant *in silico*. This showed that the variant was predicted to be tolerable with a score of 0.14 in SIFT and a score of 0.688 in PolyPhen (possibly damaging, Table 3). The location of the variants is presented in Figure 2. Considering the possible pathogenicity predicted by bioinformatic analyses, we focus on the compound heterozygous variants in particular and make a further analysis.

**Table 3:** Bioinformatics analysis of *DNAH11* variants identified by whole-genome sequencing

Patients	Positions	Locations	Base change	Amino acid change	Status	Allele frequency	HSF3	SIFT	Polyphen
P1	21784425	58	c.G9484-1T	–	He	–	Mostly probably affecting splicing	–	–
	21884331	76	c.12428T>C(rs751994566)	p.M4143T	He	0.0000747	–	Tolerated	Possibly damaging

## Discussion

Ultrastructurally, sperm flagella are fundamental to sperm motility, which has intrinsically significant associations with migration from the vagina to the Fallopian tubes, penetration of the cumulus oophorus and fertilization process during natural conception [3,17], and axonemal dynein heavy chains (DHCs) as important parts of the sperm flagella harbor the motor machinery generating the force for sliding of microtubules and powering flagellar beat [18–20]. Thus, defects in axonemal DHCs could influence dynein arm assembly and further result in PCD, including Kartagener’s syndrome and asthenozoospermia [11,21,22].

As one of the major categories of male infertility, asthenozoospermia accounts for approximately 18.71% of infertile patients with an isolated disorder and another 63.13% when combined with oligo- and/or teratozoospermia [23]. Thus, it is a serious problem causing male infertility. According to the World Health Organization, the diagnosis of asthenozoospermia is intrinsically correlated with sperm dysmotility [16]. Spermatozoa are of extraordinary complexity and any alteration in the cellular structure involved in generating the flagellar beat would result in defects in sperm motility. In particular, DHCs are in charge of generating the force for sliding microtubules and powering the flagellar beat. Because of the high degree of conservation among DHCs, related mutations can be responsible for human infertility with poor sperm motility. Furthermore, the DNAH11 protein is one of the DHC family members participating in assembling outer dynein arms, linked with the beating frequency of axoneme [11]. *DNAH11* mutations could influence beating frequency and beating amplitude, further resulting in PCD [14]. Zuccarello et al. [15] first reported a heterozygous mutation of *DNAH11* leading to isolated asthenozoospermia (sperm motility range from 2% to 16%), with no ultrastructural anomaly detected in sperm by transmission electron microscopy (TEM).

In our study, one of 87 asthenozoospermia patients was detected as carrying *DNAH11* compound heterozygous variants (c.9484-1 G>T, c.12428 T>C). The variant c.9484-1 G>T is located in the splicing acceptor site of intron 57 and predicted *in silico* to highly affect splicing. Theoretically it could cause in-frame deletions of exon 58 (38 amino acids) and truncate the coiled-coil stalk of the protein, which occurs between domains AAA4 and AAA5 and supports the ATP-sensitive microtubule binding component. This assumption could not be confirmed, but several similar splice-site mutations in *DNAH11* have been recorded. Pifferi et al. [13] reported that a splice acceptor site mutation (c.883-1G>A) could cause exon skipping by *in silico* analysis. Knowles et al. [24] also certified that a splice mutation c.2275-1G>C caused a deletion of exon 14 by cDNA analysis. The second variant M4143T (c.12428 T>C) is located at exon 76, which would have an impact on the C-terminal region for the codon 4143M is really conserved among different species, and the variant 4143T is rarely occurred and predicted to be possible damaging. Hence, we surmise *in silico* analysis that the compound heterozygous mutations (c.9484-1 G>T and c.12428 T>C) could strongly affect protein function and be pathogenically linked with low sperm motility in our case.

The limitation of the present study is that we did not perform TEM studies and could not assess the ultrastructure of sperm flagella. However, this is the first report on an association of *DNAH11* gene polymorphisms with asthenozoospermia patients in Chinese men, and could provide additional information for genetic counseling of asthenospermia patient.

## Conclusion

We found that *DNAH11* gene polymorphisms display strong associations with asthenozoospermia, and may contribute to an increased risk of male infertility in Chinese patients. Sperm ultrastructure and further mechanistic studies on the role of *DNAH11* that influence low sperm motility are suggested.

## Abbreviations

DHC: dynein heavy chain
DNAH11: axonemal heavy chain dynein type 11
FSH: follicle stimulating hormone
LH: luteinizing hormone
NP: nonprogressive motility
PCD: primary ciliary dyskinesia
PR: progressive motility
SNP: single nucleotide polymorphism
SNV: single nucleotide variant
T: testosterone
TEM: transmission electron microscopy
